# *In silico* characterization of a novel pathogenic deletion mutation identified in *XPA* gene in a Pakistani family with severe xeroderma pigmentosum

**DOI:** 10.1186/1423-0127-20-70

**Published:** 2013-09-24

**Authors:** Muhammad Nasir, Nafees Ahmad, Christian MK Sieber, Amir Latif, Salman Akbar Malik, Abdul Hameed

**Affiliations:** 1 Institute of Biomedical and Genetic Engineering, 24-Mauve area, G-9/1, Islamabad 44000, Pakistan; 2 Institute of Bioinformatics and Systems Biology, Helmholtz Center Munich-German Research Center for Environmental Health, Neuherberg, Germany; 3 Latif Clinics, Rawalpindi, Pakistan; 4 Department of Biochemistry, Quaid-i-Azam University, Islamabad, Pakistan

**Keywords:** Genetic skin disorder, Novel mutation, Nucleotide excision repair, Xeroderma pigmentosum, XPA

## Abstract

**Background:**

Xeroderma Pigmentosum (XP) is a rare skin disorder characterized by skin hypersensitivity to sunlight and abnormal pigmentation. The aim of this study was to investigate the genetic cause of a severe XP phenotype in a consanguineous Pakistani family and *in silico* characterization of any identified disease-associated mutation.

**Results:**

The XP complementation group was assigned by genotyping of family for known XP loci. Genotyping data mapped the family to complementation group A locus, involving *XPA* gene. Mutation analysis of the candidate XP gene by DNA sequencing revealed a novel deletion mutation *(c.654del A)* in exon 5 of *XPA* gene. The c.654del A, causes frameshift, which pre-maturely terminates protein and result into a truncated product of 222 amino acid (aa) residues instead of 273 *(p.Lys218AsnfsX5)*. In silico tools were applied to study the likelihood of changes in structural motifs and thus interaction of mutated protein with binding partners. *In silico* analysis of mutant protein sequence, predicted to affect the aa residue which attains coiled coil structure. The coiled coil structure has an important role in key cellular interactions, especially with DNA damage-binding protein 2 (DDB2), which has important role in DDB-mediated nucleotide excision repair (NER) system.

**Conclusions:**

Our findings support the fact of genetic and clinical heterogeneity in XP. The study also predicts the critical role of DDB2 binding region of XPA protein in NER pathway and opens an avenue for further research to study the functional role of the mutated protein domain.

## Background

Xeroderma Pigmentosum (XP) is one of the rare autosomal recessive inherited skin disorders. The incidence of the disease is 1 in 20,000 to 1 in 250,000 births in Japan and USA respectively and, approximately 2.3 per million live births in Western Europe has been reported [1,2]. XP was first described by Hebra and Kaposi in 1874 [3]. The disorder is characterized by extreme sensitivity to sunlight which leads to high incidence of skin sunburn, pigmentary changes, skin dryness and frequent neurological abnormalities. The UV exposed areas of the skin, tongue and eye have a high cancer risk [4,5]. XP can result from mutation in any one of the eight *XP* genes (*XPA-XPG and XPV*) and affects both gender, and all races across the continents [6]. Although *XP* genes have different chromosomal locations and code for eight different proteins; however, all are involved in the repair of ultraviolet (UV)-induced damages in DNA. Based on involvement of eight different genes and their protein products, XP has been sub-categorized into seven complementation groups plus one variant form (XP-A-XP-G and a variant XP-V) [7,8]. Out of eight, seven gene products (XPA-G) are required for the removal of UV-damaged part of the DNA while the eighth (XPV), a variant form, is required for the replication of DNA containing unrepaired damage [6,9]. Any pathogenic genetic change in the *XP* genes may reduce or abolish the cell stability of UV-induced DNA repair and prone them to the lethal and mutagenic effects of UV radiation damage [10]. However, severity of the disease depends on the gene involved, site of mutation and residual activity of the gene. Therefore, a wide variability in clinical features exists both between and within XP groups [6,11].

Generally, patients with XP complementation group A show the most severe clinical symptoms of skin and neurological abnormalities and in most cases, patients survive till their second or third decade [12]. Tanaka *et al.* were the first to map *XPA* gene on chromosome 9q and characterized this gene [13]. Human *XPA* gene consists of 6 exons distributed over ~25 kb of genomic DNA and encodes 273 aa. XPA is required for UV-induced DNA damage verification and confirmation that other NER proteins are in correct position before nucleases cut on either side of the damage [6].

In this study, we have identified a two generational Pakistani family suffering from XP. All the patients exhibited signs and symptoms of varying degree depending on age, sunlight exposure and elevated possibility to develop basal and squamous cells carcinoma. To find out the underlying molecular basis of XP, genetic analysis was carried out and we have identified a novel homozygous deletion mutation in exon 5 of *XPA* gene in this Pakistani family.

## Methods

### Sample collection and DNA extraction

A consanguineous Pakistani family with severe clinical skin symptoms was ascertained from Rawalpindi district, Pakistan. Three individuals, ages 2–5 years in the family were affected. Detailed clinical examination of all the family members including affected individuals and their carrier parents was carried out by a dermatologist in Rawalpindi. All the affected individuals were presented with severe clinical skin symptoms of XP. Blood samples were collected from all the affected and normal family members. Blood samples were also collected from 100 ethnically-matched unrelated normal individuals and used as a control for allele frequency calculation and confirmation of disease-associated mutation. Genomic DNA for linkage analysis was extracted from peripheral blood by standard phenol–chloroform DNA extraction procedure [14]. The study was approved by institutional ethnic committee (Ethical Committee, IB&GE, Islamabad, Pakistan) and was in concordance with the Helsinki declaration.

### Genotyping

Polymerase chain reaction (PCR)-based linkage analysis using microsatellite markers was used for the genotyping of genomic DNA of the family members. In each reaction 80 ng of genomic DNA was amplified in 10 μl final reaction volume using standard PCR protocol. Amplification was performed with an initial denaturation for 5 min at 94°C, followed by 35 cycles of denaturation at 94°C for 45 sec, annealing at 55°C for 45 sec, extension at 72°C for 45 sec and a final extension at 72°C for 10 min. The PCR product was separated on 8% non-denaturing polyacrylamide gel stained with ethidium bromide and alleles were assigned by visual inspection.

### Mutation analysis

For mutation analysis, six pairs of intronic primers were used to amplify coding DNA sequences of *XPA* gene. 250 ng of genomic DNA in 50 μl final reaction volume was amplified using standard PCR protocol. The amplification conditions were; 95°C for 5 min, followed by 35 cycles at 95°C for 45 sec, primer-specific annealing temperature for 45 sec, 72°C for 45 sec and a final extension at 72°C for 10 min. 10 μl of the PCR products were analysed on 2.5% agarose gel and remaining PCR products were purified using QIAquick PCR Purification Kit (Qiagen, U.K.) and sequenced directly using Big Dye®Terminator v3.1 cycle sequencing kit on an ABI 3130 genetic analyzer (Applied Biosystems, U.S.A.). Potential disease-associated mutation was confirmed by bidirectional sequencing, allele specific-PCR and by assessing 100 control samples having ethnic backgrounds matching the patients. Sequences were compared with the NCBI reference sequence [NG_011642.1] and sequence data from this study have been deposited in GenBank [Accession No. KC899693].

### Tetra amplification refractory mutation system (tetra ARMS-PCR)

Two inner mutation specific ARMS-PCR primer (5′-CGAGAAAAAATGAAACA GAAGAAA-3′and 5′-GAGAAAAAATGAAACAGAAGCAA-3′) were designed to verify the identified deletion. Two outer primers (5′-CATTCTTTGGTACCTTTGGA-3′ and 5′-GTAAAACACAATCCTTCACG-3′) and one inner primer were used in single reaction to amplify exon 5. ARMS-PCR reaction was carried out in 25 μl final volume containing 200 ng genomic DNA and 1 U *Taq* DNA polymerase. Amplification was performed with an initial denaturation for 5 min at 95°C, followed by 35 cycles of denaturation at 95°C for 45 sec, primer-specific annealing temperature for 45 sec, 72°C for 45 sec and a final extension at 72°C for 10 min. PCR products were analysed on 2% agarose gel and genotypes were assigned on visual examination.

### *In silico* analysis

To study the effect of deletion mutation on the coding nucleotide sequence and its impact on the protein product of *XPA* gene, CLC workbench 6.6.2 software (http://www.clcbio.com/) was used. We also used PROFsec [15] for the comparative secondary structure prediction of wild-type and mutated protein sequence in combination with ncoils [16] in order to determine coiled coil structures. The prediction of intrinsically disordered regions done by globplot [17], sections of low complexity were determined using seg algorithm [18]. Long distance interactions due to disulfide bonds that might contribute to protein structure stability were predicted by DIANNA [19]. For the localization of functional domains we scanned for Interpro [20] domains and Prosite [21] patterns.

The prediction of tertiary structure has been performed by phyre2 [22] and 3djigsaw [23] algorithms. Super-positioning of wild-type and mutated structure was done by Dalilite [24], for the visual comparison we used Pymol (http://www.pymol.org).

## Results

### Clinical observations

**Figure 1:**
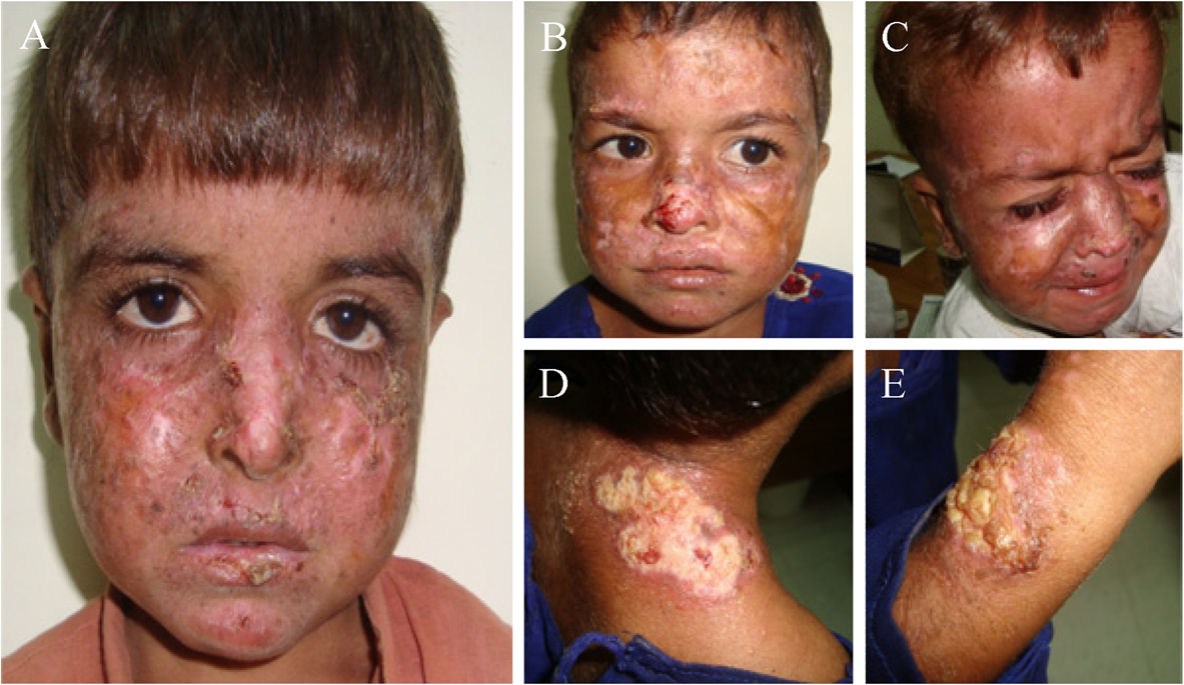
**A two generation Pakistani family suffering from severe manifestations of xeroderma pigmentosum complementation group A**. Patients ages ranging from 2–5 years **(A)** patient IV:1 in pedigree; pigmentary macules of varying sizes, atrophy, dryness, scaring, cheilitis, ulcer on nose (may possibly BCC), ulcer on lips & below left eye covered with scab & hyperkeratosis **(B)** patient IV:2 in pedigree; marked atrophy with hypo-pigmentation, dry skin pigmentary macules, ulcer with thread like irregular margin on nose with possible risk to develop BCC **(C)** patient IV:3 in pedigree; pigmentary macules, hypo-pigmentation, atrophy, dryness, ulcer above upper lips, ulcer and pigmentary changes with atrophy on right ear as well. **(D, E)** patient IV:2 in pedigree; Ulcer with irregular margin with peripheral atrophy and pigmentary changes may possibly be SCC.

**Figure 2:**
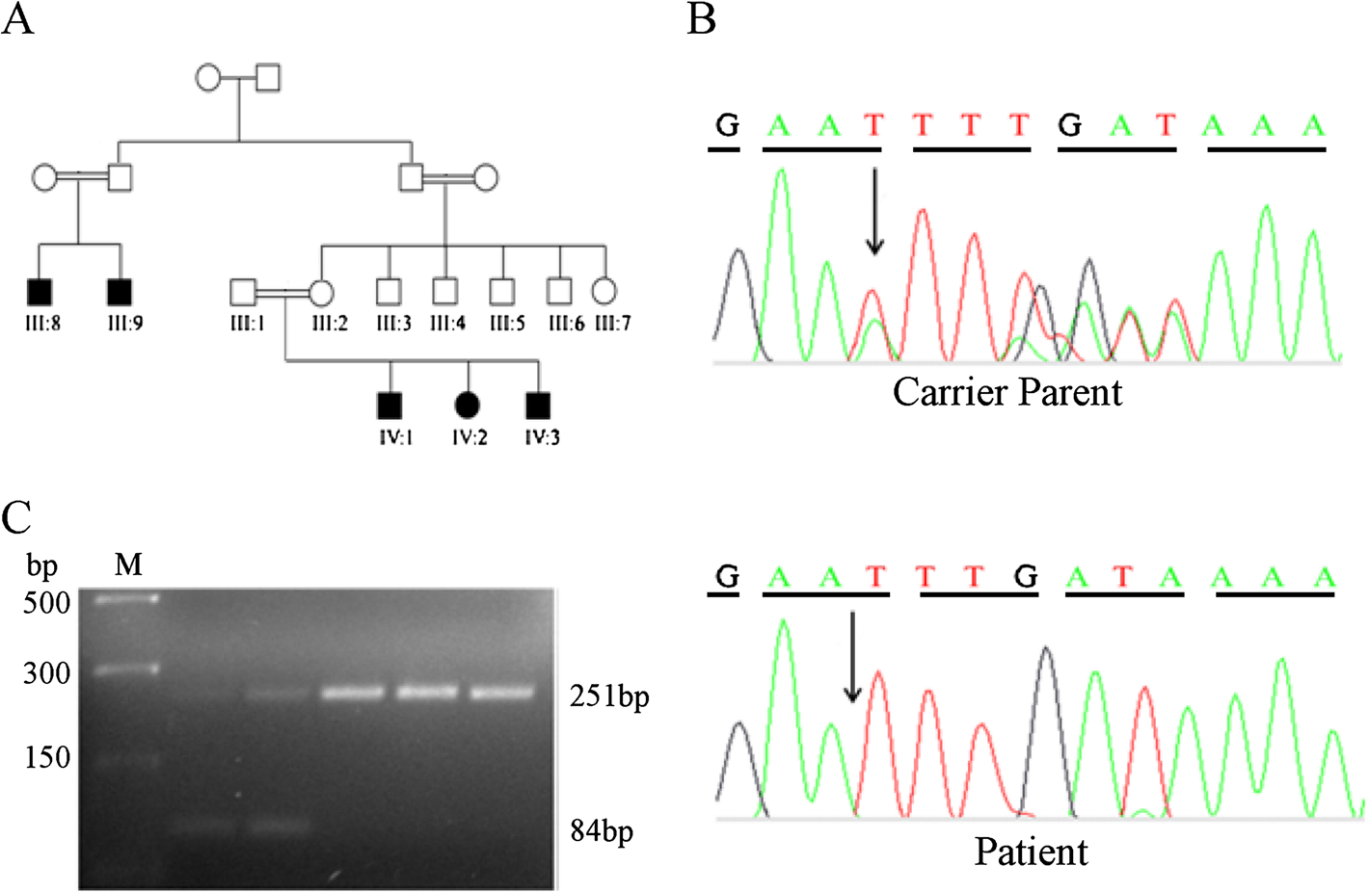
**The XP family pedigree and DNA sequence analysis. (A)** A multi-generation consanguineous Pakistani family in which XP is segregating as an autosomal recessive trait. Three affected individuals (IV-1, IV-2, IV-3) and two carrier parents (III-1, III-2 were analysed. **(B)** Direct sequencing of the PCR product amplified from exon 5 of XPA gene, revealed an adenine base deletion at position 654 (c.654 del A). The affected individuals are homozygous for this deletion (arrow), while the parents are heterozygous (carriers) having a normal allele as well as a mutant allele. **(C)** ARMS-PCR analysis also showed a heterozygous condition of both normal carrier parent and homozygous condition of all 3 affected individuals.

All the patients possibly affected by birth as the symptoms of the disease appeared in the first year of their life. The affected individuals had very severe clinical symptoms of XP that include severe sunburn, blisters, freckles, irregular pigmentary macules of varying sizes, atrophy, dryness, ulcers on different body parts with possible risk to develop basal cells carcinoma (BCC) and squamous cells carcinoma (SCC). However, no ocular and neurological involvement was observed in any of the affected individual (Figure 1). In XPA patients the appearance of more severe clinical sign and symptoms, such as neurological involvement is reported to be progressive. It is therefore, premature to say anything about the neurological involvement of the disease in patients investigated because of their young ages. Further, the patient III: 8 and III: 9 (Figure 2A), which belongs to a superior generation than the patients studied would have added more to clinical details, such as neurological manifestations of the disease. Unfortunately, both the patients were not available for inclusion in this study.

### Genotyping

Genotyping of affected family members and their carrier parents (Figure 2A) was carried out using PCR-based linkage analysis. Genotyping analysis revealed an evidence of linkage with microsatellite markers; D9S301, D9S303, D9S924, and D9S167 at 9q22.3. All the microsatellite markers mentioned above were fully informative and homozygous in all the affected individuals. The results were therefore, consistent with the recessive mode of segregation of the disease in this family. The mapped region of homozygosity at locus 9q22.3 harbors previously reported XP associated *XPA* gene [13].

### Mutation screening of *XPA* gene

Direct DNA sequencing of PCR products from exon 5 revealed a novel homozygous deletion in the *XPA* coding sequences at nucleotide position 654 *(c.654 del A)*. All the affected individuals of the family were homozygous for this deletion, whereas both the normal parents were heterozygous for the mutation (Figure 2B). However, deletion was not observed in any control samples.

ARMS-PCR using allele specific primers revealed two bands of sizes 251 bp and 84 bp in both heterozygous carrier parents while only one band was observed in all patients homozygous for the deletion mutation (Figure 2C). ARMS-PCR analysis of exon 5 of *XPA* gene showed that the mutant allele co-segregate with the disease phenotype in the family.

### Evaluation of deletion mutation

Mutation analysis using CLC workbench 6.6.2 software (http://www.clcbio.com/) revealed that adenine deletion at nucleotide 654 has altered the Lys/K-218 codon (AAA) to an Asn/N-codon (AAT) and resulted in downstream premature termination of XPA protein at amino acid 222.

### Comparative modeling of protein

A comparative analysis of sequence and structure features reveals the consequences of the point mutation in the resulting protein. Predicted secondary structures showed that the mutated amino acid is located in a coiled coil alpha helix and causes an interruption of the structure.

Alignments of Interpro- and Prosite-motifs show that the coiled coil alpha helix is part of the domain that is responsible for DNA binding. Despite the shorter structure in the mutated protein, the binding domain is located around 20 residues from the C-terminal end. The same holds for the other XPA specific domains. A main difference is one predicted cAMP- and cGMP-dependent protein kinase phosphorylation site that is only present in the wild-type c-terminal region.

**Figure 3:**
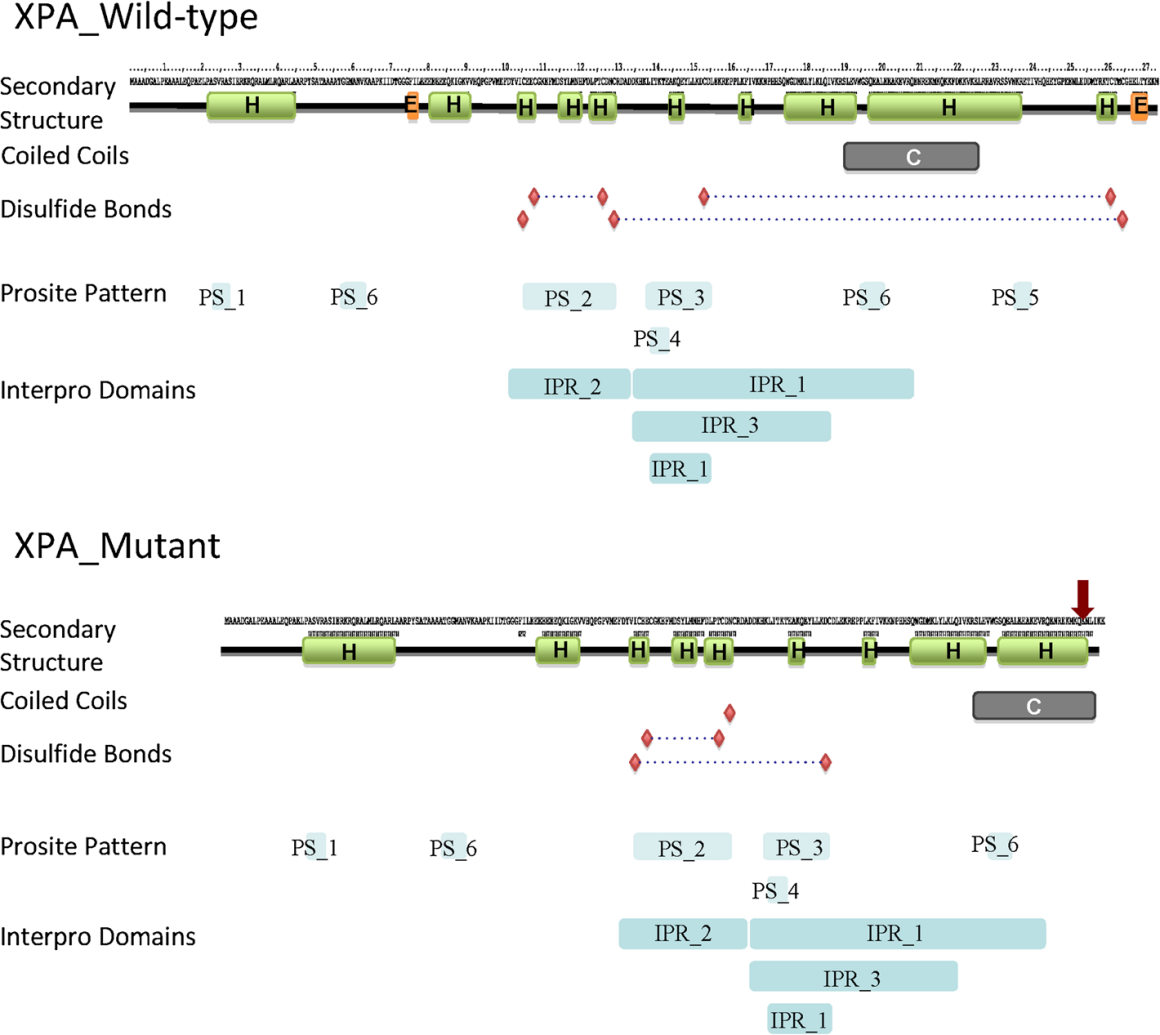
**Comparison of predicted sequence and structure features between wild-type and mutant XPA protein.** Secondary structure is depicted as green (alpha-helices) and orange (beta-sheets) boxes. Prediction of coiled coil regions is shown as grey bar. Red diamonds indicate cysteins involved in disulfide bonds, whereas dashed connections show predicted bonds between them. Blue boxes indicate regions of functional domains determined by Interpro and Prosite. Prosite Pattern: PS_1: Protein kinase C phosphorylation site, PS_2: XPA protein signature 1, PS_3: XPA protein signature 2, PS_4: Casein kinase II phosphorylation site, PS_5: cAMP- and cGMP-dependent protein kinase phosphorylation site, PS_6: N-myristoylation site. Interpro-Domains: IPR_1: DNA binding domain, putative, IPR_2: Zinc finger, XPA-type, conserved site, IPR_3: XPA-type C-terminal, IPR_4: XPA conserved site.

Furthermore, in this region two disulfide bonds are located. DIANNA detected in total three bonds in the WT structure between CYS-153, CYS-261 and CYS-129, CYS-264 as well as between CYS-108, CYS-126. A different binding pattern is predicted in the mutated structure. Here the cysteins 105 and 153 as well as 108 and 126 form disulfide bonds (Figure 3).

**Figure 4:**
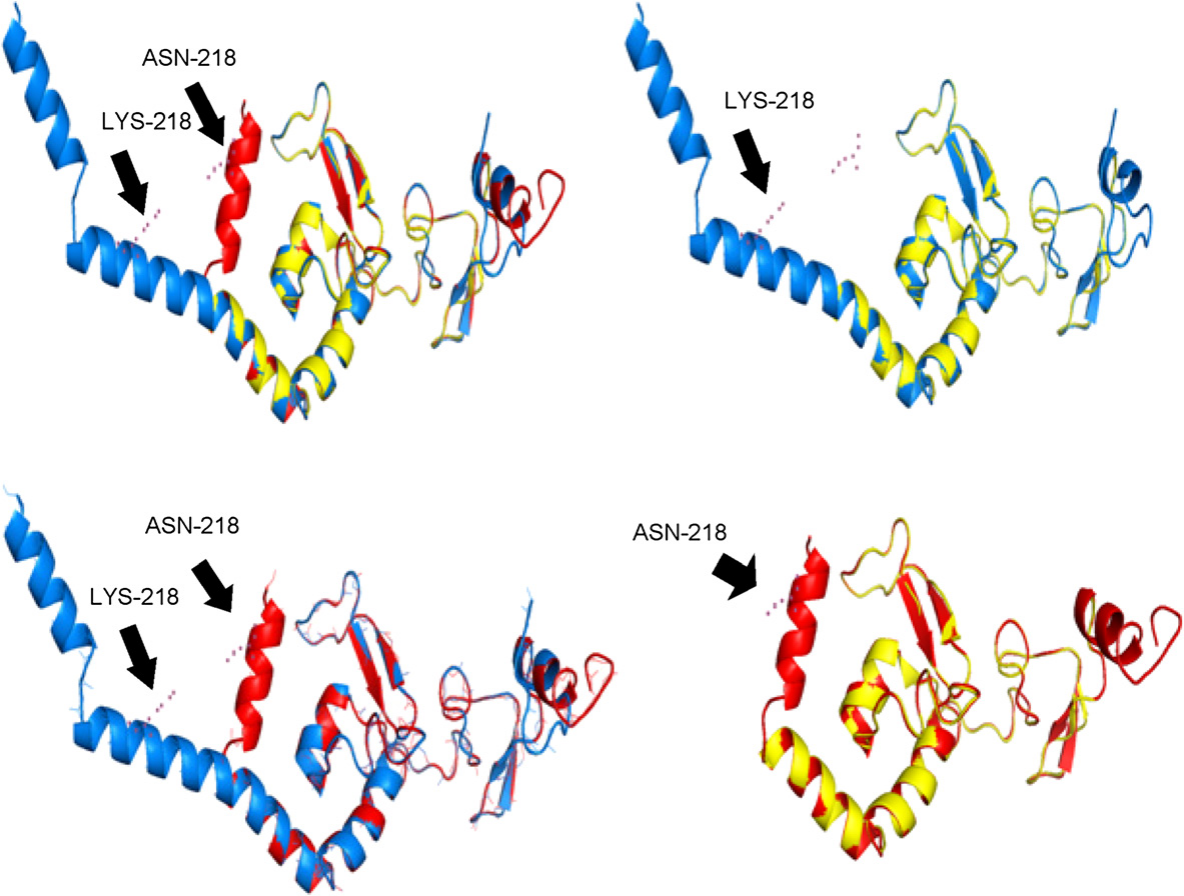
**Superimposed tertiary structure of 1XPA and predictions of wild type and mutant XPA protein.** 1XPA PDB-structure is illustrated in yellow. Predictions of the full wild-type and mutated protein is depicted in blue and red, respectively. The mutated site and its pendant in wild-type are indicated by arrows.

We utilized two algorithms for tertiary structure prediction. Both predictions show conserved structures in the core region between residues 99 and 210 while the terminal regions differ strongly from each other. However, both predictions show a difference in angle of the coiled coil alpha helix in the mutated structure compared to the wild-type. The conserved region corresponds to the experimentally determined Protein Data Bank (PDB) structure 1XPA (Figure 4).

## Discussion and conclusions

Xeroderma pigmentosum is a genetically heterogeneous skin disorder and among its eight complementation groups, XPA form is the most frequent worldwide [11]. To date, more than 30 mutations have been reported in different functional domains of *XPA* gene (Additional file 1: Table S1), which is an indicative of wide genetic variability across this gene and high level of clinical heterogeneity [12]. Several research articles have concluded that the severity of clinical symptoms depend on the site of mutation in *XPA* gene and the residual activity of the mutated XP protein. Therefore, as we move from N terminal (5′ end) towards C terminal (3′ end) the severity of clinical manifestations decreases, except in cases where splice site mutations allow synthesis of small amount of normal protein [25].

Here we studied a consanguineous Pakistani family with severe clinical symptoms of XP and identified a novel homozygous deletion mutation in exon 5 of *XPA* gene. Deletion mutation not only led to a shift in the DNA frame but also resulted in premature termination of XPA protein translation just 5 residues downstream from the point of deletion. Hence, resulted premature truncated XPA protein consists of 222 aa instead of normal 273 aa *(p.Lys218AsnfsX5)*. The presence of deletion mutation in homozygous condition in all the patients, heterozygous condition in both normal carriers and its absence in all unrelated 100 controls confirms disease association of this novel deletion with XP in Pakistani family.

Analysis of the sequences and their structural features shows that the mutation interrupts a coiled coil alpha helix that is part of the functional domain for DNA binding. According to the sequence alignment of the Interpro motif, the mutated part of the helix is not involved in the bidding process; anyhow binding affinity of the whole helix might be affected due to the interruption. The XPA-functional domains are present in both wild-type and mutant proteins and are located in the core region with the highest structure conservation compared to 1XPA structure.

Another main difference of the two structures is the predicted disulfide-binding pattern. In the wild-type protein three disulfide bonds are predicted. One bond is located in the part not affected by the mutation and can therefore be found in both proteins, whereas in the other two cysteins from the unique part are involved and thus only exist in the wild-type. The residue CYS-153 is bound in both cases but connected to different cysteins. These differences in long term interactions might affect the stability of the protein and contribute to differences in the tertiary structure.

Super-positioning of the predicted structures and the experimentally determined PDB structure 1XPA shows that the core region between residues 99 and 210 is similar in terms of three-dimensional structure whereas the C- and N-terminal parts differ strongly from each other. Explanations might be different binding pattern of disulfide bonds, or problems of the algorithm in modeling the structure due to missing templates and a lot of loop- and turn-structures in the N-terminal region. The three dimensional position of the shorter coiled coil alpha helix differs in both predictions what might also be a hint for a different binding affinity of the protein. The predicted cAMP- and cGMP-dependent protein kinase phosphorylation site, which is missing in the mutated protein might play a role in activation or inhibition of the protein.

**Figure 5:**
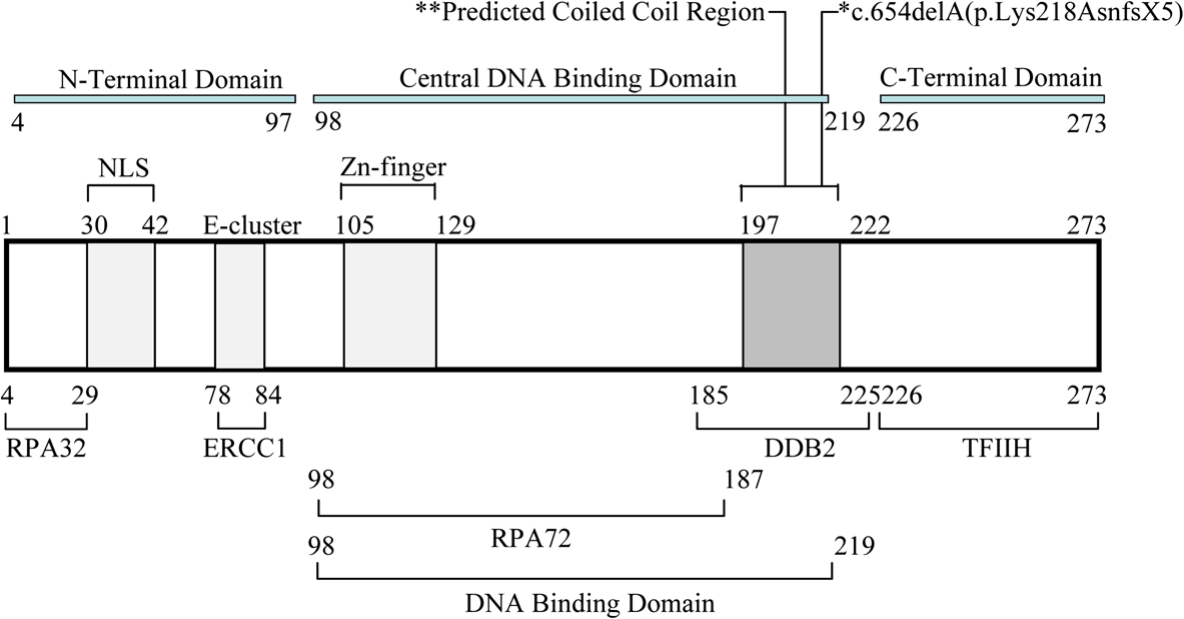
**Schematic presentation of XPA protein with distinct functional domains.** Regions for binding to RPA, ERCC1, DDB2, TFIIH and location of the nuclear localization signal (NLS) and the zinc finger motif are shown. Numbers refer to amino acid numbers for the XPA protein. *Deletion mutation identified in this study. ** Predicted Coiled coil structure formation region.

To establish genotype/phenotype correlation of XP in Pakistani family, different functional regions of XPA and its interacting proteins were considered. Different domains of XPA play a unique role in NER reaction by interacting with Replication protein A (RPA), Excision repair cross complementing group 1(ERCC1), DNA damage-binding protein 2 (DDB2), transcription factor II H (TFIIH) and as well as to UV- or chemical carcinogen-damaged DNA [26,27]. Our results clearly show that the deletion identified is present in the central DNA binding domain of the XPA that span from residues M98-F219 (Figure 5). DNA binding domain is one of the major functional regions of XPA protein that preferentially bind to the damaged DNA [28,29]. Mutation in this crucial region might have altered the binding capacity of XPA protein to the damaged DNA, as predicted by using *in silico* tools. Secondly, among the interacting proteins DDB2 plays a key role in DNA repair by making physical interaction with residues 185–226 in XPA and initiate DDB-mediated NER reaction (Figure 5). Changes in this region has been reported to decrease the interaction between XPA and DDB2 [28,30]. Therefore, we can suggest that nucleotide deletion we found in this region might have altered the interaction between XPA and DDB2 and, resulted in inability of cells to carry out efficient repairing of UV-induced DNA damages. Third aspect of the condition, where deletion found in codon 218 causes the termination of translation at codon 222 that resulted in complete elimination of exon 6 from the translated protein (Figure 5). Exon 6 interacts with transcription factor TFIIH, a component of NER complex formed at DNA damaged site, and play an important role in DNA repair [28]. Therefore, possible explanation for severe clinical manifestations of XP in Pakistani family, other than prolonged sunlight exposure, might be the result of complete loss of exon 6 function or deletion mutation in exon 5 itself might have altered or completely abolished the capability of XPA protein to interact with damaged DNA and DDB2 protein.

In a nutshell our results suggest that the 3′ half of exon 5 from 207–222aa may play an important accessory role in DNA binding and retain a significant and clinically important residual function in DNA repair. The expression studies of mRNA and protein in clinical samples of patients would have been supportive to assure the relevance of *in silico* predictions of mutant protein and its correlation with clinical manifestation of the disease. Unfortunately, the fresh clinical samples were not available any more to conduct expression and functional studies as mentioned in the results section.

## Abbreviations

XP: Xeroderma pigmentosum; NER: Nucleotide excision repair; DDB2: DNA damage-binding protein 2; ARMS-PCR: Amplification refractory mutation system-polymerase chain reaction; ERCC1: Excision repair cross complementing group 1; RPA: Replication protein A; TFIIH: Transcription factor II H; PDB: Protein Data Bank; BCC: Basal cell carcinoma; SCC: Squamous cell carcinoma.

## Competing interests

The author(s) declare that they have no competing interests.

## Authors’ contributions

MN designed the study, performed experimental work, paper writing and drafting, NA participated in experimental work and paper drafting, CMKS provided protein modeling expertise and paper writing, AL provided clinical expertise, SAM participated in paper drafting and assisted in study design. AH analyzed data and assisted in manuscript preparation. All authors read and approved the final manuscript.

## Supplementary Material

Additional file 1: Table S1 *XPA* gene reported so far. Click here for file
