# Supplementary material for: In silico characterization of a novel pathogenic deletion mutation identified in XPA gene in a Pakistani family with severe xeroderma pigmentosum
Source: J Biomed Sci. 2013 Sep 24;20(1):70. doi: 10.1186/1423-0127-20-70 (PMC3856591; doi:10.1186/1423-0127-20-70)
Supplement: Additional file 1: Table S1 — XPA gene reported so far. [file 1423-0127-20-70-S1.docx]

**Additional Table 1**. List of the mutations in *XPA* gene reported so far;

| **Position** | **Sequence**  **Change** | **Mutation Type** | **Amino Acid  Change** | **Patient Origin/**  **Ethinicity** | **Reference** |
| --- | --- | --- | --- | --- | --- |
| Exon 2 | c.281 C>T | Missense | p.P94L | Japanese | Tanaka (1993)  *Jpn J Hum Genet* 38:1 |
| Exon 3 | c.323G>T | Missense | p.C108F | Caucasian | Satokata (1992) *Hum Genet* 88:603 |
| Exon 3 | c.331G>T | Nonsense | p.E111X | Tunisian | Messaoud (2012) *Arch Dermatol Res* 304:171 |
| Exon 3 | c.348T>A | Nonsense | p.Y116X | Japanese | Satokata (1992) *Mutat Res/DNA Repair* 273:193 |
| Exon 3 | c.377 G>A | Missense | p.C126Y | American and European | States (1998) *Hum Mutat* 12:103 |
| Exon 5 | c.619C>T | Nonsense | p.R207X | Palestinian | Satokata (1992) *Mutat Res/DNA Repair* 273:193 |
| Exon 5 | c.622C>T | Nonsense | p.Q208X | Japanese | Maeda (2000) *Br J Dermatol* 143:174 |
| Exon 5 | c.631C>T | Nonsense | p.R211X | Caucasians | Satokata (1992) *Mutat Res/DNA Repair* 273:203 |
| Exon 6 | c.683G>A | Missense | p.R228Q | United States | Porter (2005) *DNA Repair*  4:341 |
| Exon 6 | c.682C>T | Nonsense | p.R228X | Japanese | Satokata (1992) *Mutat Res/DNA Repair* 273:193 |
| Exon 6 | c.700G>T | Missense | p.V234L | United States | Porter (2005) *DNA Repair*  4:341 |
| Exon 6 | c.731A>G | Missense | p.H244R | Caucasians | Satokata (1992) *Mutat Res/DNA Repair* 273:203 |
| Intron 1 | c.172+2T>G | Splice site |  | Japanese | Tanioka (2005) *J Invest Dermatol* 125:244 |
| Intron 3 | c.389G>A | Splice site |  | Black | Satokata (1992) *Mutat Res/DNA Repair* 273:203 |
| Intron 3 | c.390-12A>G | Splice site |  | Mediterranean Region | States (1998) *Hum Mutat* 12:103 |
| Intron 3 | c.390-2A>G | Splice site |  | Caucasian | Satokata (1995) *Hum Mol Genet* 4:1993 |
| Intron 3 | c.390-1G>C | Splice site |  | Japanese | Tanaka (1990) *Nature* 348:73 |
| Intron 3 | c.390-1G>T | Splice site |  | Caucasian | Satokata (1995) *Hum Mol Genet* 4:1993 |
| Intron 4 | c.555G>C | Splice site |  | Caucasians | Satokata (1992) *Mutat Res/DNA Repair* 273:203 |
| Intron 4 | c.555+8A>G | Splice site |  | Sub-continent  (Punjab) | Sidwell (2006) *Br J Dermatol* 155:81 |
| Intron 5 | c.673G>C | Splice site |  | Japanese | Sato (1996) *Mutat Res* 362:199 |
| 5' UTR | c.(-4)A>G | Polymorphism |  | European Caucasians | Butkiewicz (2004) *Cancer Epidemiol Biomarkers Prev* 13: 2242 |
| Exon 3 | c.288delT | Deletion | Frame shift | United States | Christen-Zaech (2009) *Arch Dermatol* 145:1285 |
| Exon 3 | c.349-353del5 | Deletion | Frame shift | Caucasian | Satokata (1992) *Hum Genet* 88:603 |
| Exon 3 | c.374delC | Deletion | Frame shift | Caucasian/ Egypt | Satokata (1992) *Hum Genet* 88:603 |
| Exon 4 | c.467_486del 20 | Deletion | Frame shift | Dutch | Cleaver (1995) *Hum Mol Genet* 4:1685 |
| Exon 4 | c.469_472delAA | Deletion | Frame shift | Mediterranean Region | States (1998) *Hum Mutat* 12:103 |
| Exon 5 | c.647_648del2 | Deletion | Frame shift | American and  European | States (1998) *Hum Mutat* 12:103 |
| Exon 5 | c.660_666insA | Insertion | Frame shift | Dutch | Cleaver (1995) *Hum Mol Genet* 4:1685 |
| Exon 6 | c.689_690insT | Insertion | Frame shift | Japanese | Takahashi (2010) *J Invest Dermatol* 130:2481 |
| Exon 6 | c.721_722insG | Insertion | Frame shift | Tunisian | Messaoud (2010) *Br J Dermatol* 162:883 |
| Exon 6 | c.778_779insTT | Insertion | Frame shift | Japanese | Takahashi (2010) *J Invest Dermatol* 130:2481 |
| Exon 6 | c. 779_780insTT | Insertion | Frame shift | Japanese | Takahashi (2010) *J Invest Dermatol* 130:2481 |
